# Cbl-b predicts postoperative survival in patients with resectable pancreatic ductal adenocarcinoma

**DOI:** 10.18632/oncotarget.18714

**Published:** 2017-06-27

**Authors:** Qian Dong, Yuteng Ma, Yao Zhang, Xiujuan Qu, Zhi Li, Yafei Qi, Yunpeng Liu, Ce Li, Kai Li, Xianghong Yang, Xiaofang Che

**Affiliations:** ^1^ Department of Oncology, Shengjing Hospital of China Medical University, Shenyang 110004, China; ^2^ Department of Gastrointestinal Surgery, Peking University Cancer Hospital, Beijing 100142, China; ^3^ Department of Ultrasound, Shengjing Hospital of China Medical University, Shenyang 110004, China; ^4^ Department of Medical Oncology, The First Hospital of China Medical University, Shenyang 110001, China; ^5^ Department of Pathology, Shengjing Hospital of China Medical University, Shenyang 110004, China

**Keywords:** pancreatic ductal adenocarcinoma (PDAC), Cbl-b, prognosis

## Abstract

Casitas B-lineage lymphoma b (Cbl-b) is a ubiquitin-protein ligase and a signal transducing adaptor protein involved in immune regulation, and it may be involved in the development and progression of cancer. We investigated the association between Cbl-b expression and prognosis in patients with resectable pancreatic ductal adenocarcinoma (PDAC). The clinicopathological characteristics and survival data of 134 patients with surgery for PDAC between January 2009 and February 2012 were retrospectively evaluated, and Cbl-b expression was assayed by immunohistochemical staining. The association of Cbl-b expression with clinicopathological features and postoperative prognosis was analyzed. Cbl-b expression was strongly associated with the pathological primary tumor (pT) category (*P* = 0.005) and pathological TNM (pTNM) stage (*P* = 0.035), but not with other clinicopathological characteristics (all *P* > 0.05). In addition to current markers including pathological regional lymph nodes (pN) category, CA19-9, and histological differentiation, univariate and multivariate analysis found that Cbl-b was independently associated with overall survival (OS) of patients with resectable PDAC. Cbl-b was predictive of OS in a subgroup of patients with serum CA19-9 ≥ 37 U/mL. Cbl-b expression combined with pN, histological differentiation, and CA19-9 level could be used as a novel clinical model predictive of OS for patients with resectable PDAC. In conclusion, Cbl-b in resectable PDAC was an independent predictor of adverse prognosis. Cbl-b expression together with pN, histological differentiation, and CA19-9 level might lead to improved risk stratification and prognosis for patients with resectable PDAC.

## INTRODUCTION

Despite advances in treatment, the prognosis of pancreatic cancer is dismal, and the 5-year survival rate is < 7% [1]. In China, the cancer statistics show that the 5-year survival rate of pancreatic cancer is 4.1% and the median survival time is only 3.9 months [2]. For patients with pancreatic cancer, surgical resection is the only curative treatment, but fewer than 20% of patients are indicated for radical surgery, and their 5-year survival rate is only 10%–25%[3, 4]. It is very important to accurately predict the prognosis after tumor resection for the assessment of therapeutic effect, choice of adjuvant therapy, and informing patients.

To date, various biomarkers have been reported to be the prognostic markers for pancreatic cancer. Among them, the serum carbohydrate antigen 19-9 (CA19-9) level is the most widely accepted tumor marker for pancreatic cancer. It has been reported that the CA19-9 level can be used in the diagnosis, the evaluation of resectability, monitoring the progression and the prognostic prediction of pancreatic cancer [5–7]. In addition, some new prognostic markers, such as PD1/PD-L1 [8], non-coding RNAs [9] and circulating tumor DNA [10], have been reported recently. However, it may be more meaningful to identify prognostic markers from molecules involved in the malignant biological behavior of pancreatic cancer. Despite being a hypovascular malignant tumor, pancreatic cancer cells are still capable of rapid proliferation. The strong proliferative capacity significantly affects the prognosis of patients. Casitas B-lineage lymphoma b (Cbl-b) is a member of the ubiquitin ligase casitas B (Cbl) family and acts as a ubiquitin-protein ligase and adaptor protein [11, 12]. We previously showed that silencing Cbl-b expression activated the Smad3/p21 axis and inhibited proliferation of pancreatic ductal adenocarcinoma (PDAC) cells [9]. However, whether Cbl-b can be used as a prognostic marker for pancreatic cancer remains unknown. So far, studies of the association between Cbl-b and prognosis in malignancies are rare[13–15]. Li et al reported that presence of the rs2305035, a variant AA or AG genotypes was associated with overall survival (OS) [16], and patients with Cbl family mutations (e.g., c-Cbl, Cbl-b, and Cbl-c) for myeloid malignancies had poor prognosis [17]. However, there is no relevant research about the prognostic value of Cbl-b expression in PDAC patients. If a relationship between Cbl-b expression and the prognosis in PDAC does exist, then interfering with Cbl-b expression or the Cbl-b signal pathway may be able to prolong survival of PDAC patients, and thus shed light on potential therapeutic targets and prognostic biomarkers.

The study aim was to determine the prognostic value of Cbl-b in patients with PDAC. The expression of Cbl-b in human PDAC tissues was evaluated by immunohistochemistry, and its association with clinical outcomes was evaluated. The results may improve our understanding of the clinical significance of Cbl-b in PDAC and its prognostic value in PDAC patients.

## RESULTS

### Demographic and clinicopathological characteristics

The demographic and clinical characteristics of the 134 included patients are shown in Table 1. The median age was 60 years (P_25_–P_75_, 54–68 years; range, 35–80 years), and 78 (58.2%) were men. The median tumor size was 4.0 cm (range, 1.2–10 cm), 113 patients (84.3%) had well or moderately differentiated tumors, 21 (15.7%) had poorly differentiated tumors, 130 (97.0%) had negative surgical margins, and 42 (31.3%) had lymph node metastasis. Sixty-nine (51.5%) patients had tumors that extended beyond the pancreas. 3 (2.2%) had vascular tumor thrombi, and 33 (24.6%) had invasion of adjacent organs. At the last follow-up, 112 patients (83.6%) had died. Median OS was 15.9 months [95% confidence interval (CI): 13.0–18.7] (Supplementary Figure 1).

**Table 1:** The relationship of Cbl-b expression and clinicopathological characteristics in 134 patients with resectable pancreatic ductal adenocarcinoma

Characteristics	No. of cases (n, %)	Cbl-b expression		χ^2^	*P* value
		Low	High		
Age (years)*					
<60	65(48.5)	18(40.9)	47(52.2)	1.514	0.218
≥60	69(51.5)	26(59.1)	43(47.8)		
Sex					
Male	78(58.2)	30(68.2)	48(53.3)	2.678	0.102
Female	56(41.8)	14(31.8)	42(46.7)		
Maximum tumor diameter (cm)^#^					
<4.0	57(42.5)	17(38.6)	40(44.4)	0.408	0.523
≥4.0	77(57.5)	27(61.4)	50(55.6)		
Differenciation					
well/ moderately	113 (84.3)	39 (88.6)	74 (82.2)	0.920	0.337
poor	21 (15.7)	5 (11.4)	16 (17.8)		
Surgical margins					
Negative	130(97.0)	42(95.5)	88(97.8)	-	0.597^&^
Positive	4(3.0)	2(4.5)	2(2.2)		
pT category					
pT1+pT2	65(48.5)	29(65.9)	36(40.0)	7.942	0.005
pT3+pT4	69(51.5)	15(34.1)	54(60.0)		
pN category					
pN0	92(68.7)	33(75.0)	59(65.6)	1.225	0.268
pN1	42(31.3)	11(25.0)	31(34.4)		
Vascular tumor thrombus					
No	131(97.8)	43(97.7)	88(97.8)	-	1.000^&^
Yes	3(2.2)	1(2.3)	2(12.2)		
Adjacent organs invasion					
No	101(75.4)	37(84.1)	64(71.1)	2.682	0.101
Yes	33(24.6)	7(15.9)	26(28.9)		
pTNM category					
I	53(39.6)	23(52.3)	30(33.3)	4.434	0.035
II /III	81 (60.4)	21 (47.7)	60 (66.7)		
CA19-9 (U/mL)*					
< 37	18 (15.5)	5 (13.2)	13 (16.7)	0.240	0.624
≥ 37	98 (84.5)	33 (86.8)	65 (83.3)		

*Age (years): Median (P_25_-P_75_): 60 (54-68).

^#^ Maximum tumor diameter (cm): Median (P_25_-P_75_): 4.0 (3.0-5.63).

^&^ Fisher’s exact test.

** Of the 134 patients included in the present study, the values of serum CA19-9 were available for 116 patients. There were 18 missing values for CA19-9.

pT: pathological primary tumor; pN: pathological regional lymph nodes; pTNM: pathological TNM.

### Association of Cbl-b expression and clinicopathological characteristics

**Figure 1:**
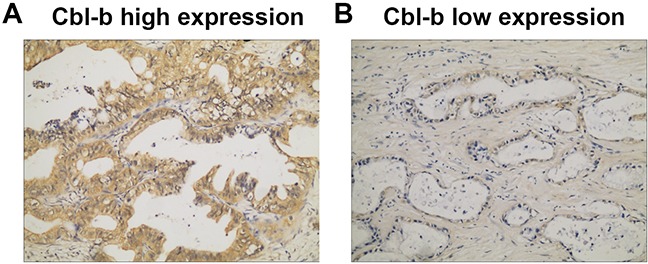
Cbl-b expression in PDAC tissue assayed by immunohistochemistry. Positive expression is shown by brown-yellow particles distributed in the cell membrane and cytoplasm (SP staining ×200) **(A)** Strong Cbl-b high expression. **(B)** Weak Cbl-b expression.

Cbl-b protein expression was assayed by immunohistochemistry, and positively stained cells were identified by the presence of brown-yellow particles located in the membrane and cytoplasm (Figure 1). Cbl-b expression in PDAC tissues varied, and was strongly associated with pT (*P* = 0.005) and pTNM stage (*P* = 0.035), but was not correlated (all *P* > 0.05) with other clinicopathological characteristics (Table 1).

### Univariate and multivariate analysis of prognostic factors for overall survival

**Figure 2:**
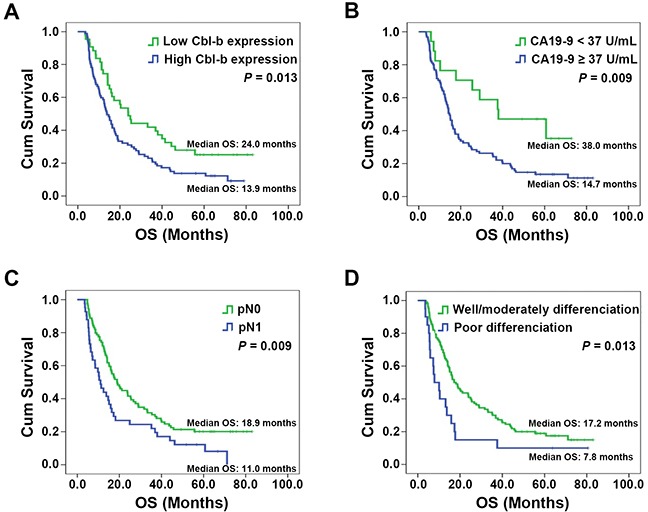
Overall survival (OS) in patients stratified by prognostic factors found to be independently associated with OS (**(A)** Cbl-b expression; **(B)** Serum CA19-9 levels; **(C)** pN category; **(D)** Histological differentiation). Significant differences in OS were revealed by the log-rank test.

**Figure 3:**
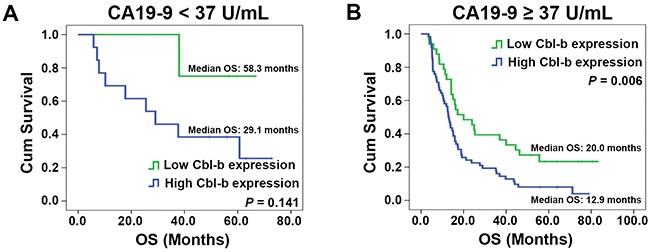
Overall survival (OS) in patients with different CA19-9 levels according to Cbl-b expression (**(A)** CA19-9 <37 U/mL; **(B)** CA19-9 ≥37 U/mL). Significant differences were revealed by the log-rank test.

The log-rank test results are shown in Table 2. Age (*P* = 0.049), histological differentiation (*P* = 0.013), pN category (*P* = 0.009), pTNM stage (*P* = 0.011), serum CA19-9 level (*P* = 0.009), and Cbl-b expression (*P* = 0.013) were significantly associated with OS. In univariate analysis, other characteristics (sex, maximum tumor diameter, surgical margins, pT category, vascular tumor thrombus, invasion of adjacent organs) did not influence OS (all *P* > 0.05). The multivariate Cox proportional hazards model (forward selection) was fitted using all 12 clinical and pathological variables, and found that high Cbl-b expression (HR, 2.048; 95% CI: 1.285–3.266; *P*=0.003), serum CA19-9 level ≥37 U/mL (HR, 2.765; 95% CI: 1.424–5.369; *P*=0.003), lymph node metastasis (HR, 1.713; 95% CI: 1.112–2.638; *P*=0.015), and poor tumor differentiation (HR, 2.299; 95% CI: 1.354-3.905; *P*=0.002) were independently associated with the poor OS (Table 3). As shown in Figure 2, patients with increased Cbl-b expression, increased CA19-9 level, lymph node metastasis, or poor tumor differentiation had shorter OS and that the OS in sub-group of serum CA19-9 ≥37 U/mL was significantly associated with Cbl-b expression (Figure 3). These data suggested that, in addition to currently used markers (pN category, CA19-9 level, and histological differentiation), Cbl-b has prognostic value in patients with resectable PDAC.

**Table 2:** Univariate analysis of overall survival (OS) and clinicopathological characteristics in 134 patients with resectable pancreatic ductal adenocarcinoma

Characteristics	Median OS (months) (95% CI)	Log-rank *x*^2^	*P* value
Age (years)			
< 60	14.2 (11.4–17.0)	3.874	0.049
≥ 60	18.1 (9.2–27.0)		
Sex			
Male	15.0 (11.3–18.7)	0.327	0.568
Female	17.4 (13.6–21.2)		
Maximum tumor diameter (cm)			
< 4.0	25.3 (13.6–37.1)	3.504	0.061
≥ 4.0	13.2 (9.7–16.6)		
Differenciation			
well/ moderately	17.2 (13.4–21.1)	6.162	0.013
poor	7.8 (2.1–13.5)		
Surgical margins			
Negative	15.9 (13.3–18.4)	0.324	0.569
Positive	24.9 (5.6–44.2)		
pT category			
pT1+pT2	23.7 (14.7–32.8)	3.502	0.061
pT3+pT4	14.1 (11.6–16.7)		
pN category			
pN0	18.9 (14.0–23.8)	6.815	0.009
pN1	11.0 (7.7–14.4)		
Vascular tumor thrombus			
No	16.2 (13.6–18.9)	0.051	0.821
Yes	13.7 (0.1–27.4)		
Adjacent organs invasion			
No	16.4 (12.7–20.1)	0.686	0.408
Yes	15.1 (12.3–17.8)		
pTNM stage			
I	25.3 (16.2–34.4)	6.470	0.011
II/III	13.2 (9.9–16.4)		
CA19-9 (U/mL)			
< 37	38.0 (6.1–69.9)	6.911	0.009
≥ 37	14.7 (12.5–16.8)		
Cbl-b expression			
Low	24.0 (17.1–30.9)	6.167	0.013
High	13.9 (10.7–17.0)		

pT: pathological primary tumor; pN: pathological regional lymph nodes; pTNM: pathological TNM.

**Table 3:** Multivariate analysis of prognostic factors independently associated with overall survival (OS) in patients with pancreatic ductal adenocarcinoma treated by surgical resection

Characteristics	Category	Hazard ratio	95% CI	*P* value
Cbl-b expression	high vs. low	2.048	1.285–3.266	0.003
CA19-9 (U/mL)	≥ 37 vs. < 37	2.765	1.424–5.369	0.003
pN category	Yes vs. No	1.713	1.112–2.638	0.015
Histological differentiation	poor vs. well/moderate	2.299	1.354–3.905	0.002

The multivariate Cox proportional hazards model (forward selection) was fitted using all of the clinical and pathological variables, which included age, gender, maximal tumor diameter, histological differentiation, surgical margins, pT category, pN category, vascular tumor thrombus, invasion of adjacent organs, pTNM stage, serum CA19-9 levels, and tissue Cbl-b expressions.

### Predictive nomograms for OS

**Figure 4:**
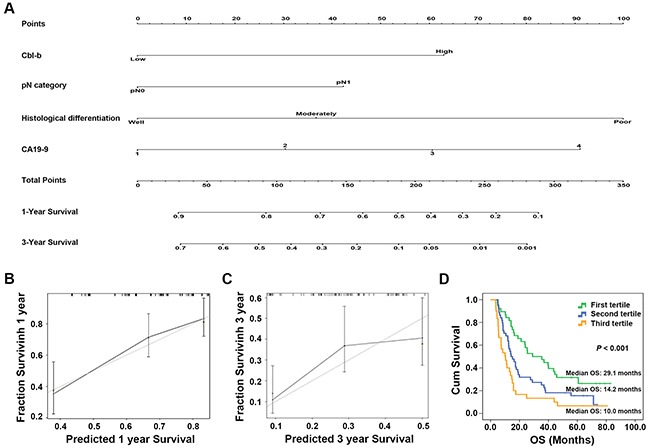
Prognostic nomogram for predicting overall survival (OS) in patients with pancreatic ductal adenocarcinoma (PDAC) treated by surgical resection Prognostic nomogram **(A)** and calibration curve **(B-C)** of Cbl-b expression combined with histological differentiation, pN category, and serum CA19-9 levels in patients with resectable PDAC. *For the nomogram, the values of CA19-9 were devided into 4 groups: CA19-9 <37 U/mL; the values of CA19-9 ≥37 U/mL was also divided into another 3 groups: the CA19-9 values were sorted from low to high, and the values of 33% and 67% were used as the cutoff points. **(D)** Kaplan-Meier curves for the OS in patients grouped according to the tertiles of the scores calculated by the prognostic nomogram. Each group represented a different prognosis (log-rank *x*^2^=16.596, *P* < 0.001).

To develop an intuitive and quantitative method of stratifying patients by prognosis, nomograms predicting 1- and 3-year OS were developed from the statistical models (Figure 4A). The predictors, including Cbl-b expression, pN category, histological differentiation, and CA19-9 level, were all independently associated with OS in multivariable analysis. Each predictor in the nomogram was weighted a number of points, and the total points for each patients was in accordance with a special predicted 1- and 3-year OS. Higher scores indicated worse prognosis (Figure 4A). The model showed good accuracy for predicting overall survival rate of PDAC treated by surgical resection, with a Harrell’s concordance index (C-index) of 0.680. Calibration curves for the nomogram predicted 1-year and 3-year OS consistent with the ideal model (Figure 4B–4C). The patients were divided into three subgroups according to the tertiles of the scores calculated by the prognostic nomogram. Kaplan-Meier curves for the OS showed that each group had a different prognosis (log-rank *x*^2^=16.596, *P* < 0.001) (Figure 4D).

In addition, to investigate whether the combination of Cbl-b expression with the traditional variables (including pN category, histological differentiation and CA19-9 level) could improve the predictive accuracy for PDAC, the C-index or Akaike information criterion (AIC) of each predictive model was compared. As shown in Table 4, the C-index of Cbl-b expression model and the traditional variables model was 0.572 and 0.666, respectively, but improved to 0.680 when Cbl-b expression was combined with the traditional variables. Similarly, the AIC of Cbl-b expression model and the traditional variables model was 751.641 and 741.623, respectively, and decreased to 732.959 when the Cbl-b expression was combined with the traditional variables. The data indicated that the combination of Cbl-b expression with pN category, histological differentiation and CA19-9 level could achieve a more reliable and precise prognostic prediction for OS of resectable PDAC patients.

**Table 4:** Comparison of the prognostic accuracies of different models

Model	C-index	AIC
Cbl-b	0.572	751.641
pN + Differentiation + CA19-9	0.666	741.623
Cbl-b + CA19-9 + pN + Differentiation	0.680	732.959

pN: pathological regional lymph nodes; C-index: Harrell’s concordance index; AIC: Akaike information criterion.

## DISCUSSION

To date, various clinicopathological features and biological markers have been reported to be prognostic factors in patients with resectable PDAC, including histological differentiation, pN category, and serum CA19-9 level [6, 18–20]. All are useful for guiding standard clinical management and establishing individualized treatment plans. In a previous study, we found that silencing of Cbl-b expression inhibited proliferation in PDAC cells *in vitro* [9]. In this study, we investigated the prognostic value of Cbl-b by retrospectively evaluating the relationship of Cbl-b expression with the clinicopathological characteristics and survival of PDAC patients. A statistically significant association was found between Cbl-b expression and survival, showing that Cbl-b level has prognostic value in patients with resectable PDAC.

Cbl-b consists of an N-terminal tyrosine kinase binding (TKB) domain, linker, RING finger domain, C-terminal proline-rich region, ubiquitin associated domain, and a leucine zipper region [21]. The specificity of Cbl-b binding to substrate proteins is determined by the recognition of specific phosphorylated tyrosine residues by the TKB domain. The RING finger has intrinsic E3 ligase activity and mediates the transfer of ubiquitin to substrates. Therefore, Cbl-b has dual functions as a ubiquitin-protein ligase and an adaptor protein [12, 22, 23]. In the immune system, Cbl-b can inhibit activation of both T CD8+ cells and natural killer (NK) cells, thereby inhibiting the inherent antitumor immune response and accelerating tumor development and progression [24]. The genetic deletion or functional inactivation of Cbl-b in NK cells can significantly inhibit the proliferation and metastasis of melanomas [25], and knockout of c-Cbl, a homologue of Cbl-b, can inhibit the proliferation of prostate cancer cells [26]. Cbl-b has also been shown to promote the proliferation of breast cancer cells [27]. We previously showed that knockdown of Cbl-b significantly decreased the proliferative activity of PDAC cells and that expression of Cbl-b and Ki-67 protein were positively correlated in PDAC tissue [9]. In this study, univariate and multivariate analysis found that Cbl-b expression had prognostic value, and was independently associated with OS of resectable PDACs.

The function of Cbl-b in tumor cells is controversial. As a negative regulator of growth factor receptor signaling and the suppression of cancer cell proliferation, Cbl-b is regarded as having anti-tumor activity. 32D/EGFR cells overexpressing Cbl-b have a markedly reduced proliferative response to epidermal growth factor (EGF), and an increased apoptosis rate [28]. Cbl-b knockdown could promote cell proliferation and reduce apoptosis induced by 5-fluorouracil treatment in gastric cancer cells via the EGFR pathway [29]. Other studies have also indicated that Cbl-b promotes the proliferation of cancer cells. In breast cancer, Cbl-b binds to the proline rich region of Smad3 and prevents the protein from translocating into the nucleus to inhibit the transcription of tumor suppressor genes downstream of the TGF-β pathway, including p21^Cip1^ and p15^INK4b^ [27]. We previously showed that silencing of Cbl-b expression inhibited proliferation in PDAC cells by up-regulation of Smad3/p21 signaling [9]. The diversity of Cbl-b substrates in cancer cells and the regulation of pathways in a cell type-dependent manner may account for differences in the effect of Cbl-b expression on proliferation of cancer cells.

This study demonstrated that serum CA19-9 levels ≥37 U/mL, poor histological differentiation, and lymph node metastasis was significantly associated with poor OS, which is consistent with previous studies [6, 18–20]. Cbl-b had significant prognostic value in a subgroup of patients with serum CA19-9 ≥37 U/mL, indicating shorter OS in patients with serum CA19-9 ≥37 U/mL and tumors with high Cbl-b expression. These findings suggested that combination Cbl-b expressions and CA19-9 levels might provide more information for prognostic prediction of PDACs. For the sub-group of serum CA19-9 <37 U/mL, the median OS of patients with Cbl-b low expression was longer than that with Cbl-b high expression, but no statistical significance was observed (*P* = 0.141). It is probably because that there were only 18 patients in the low CA19-9 group vs. 98 in the high CA19-9 group. The groups were very different in size and therefore it is difficult to draw conclusions from the low CA19-9 group. Prospective validation, including an adequate number of patients, is required to clarify the relationship between the CA19-9 level and Cbl-b expression in PDAC. Nomograms and calibration graphs had a C-index of 0.680, which supports the value of Cbl-b to predict 1-year and 3-year survival rates. Nevertheless, this study had limitations much like those of other retrospective studies. Consequently, results require validation in a large, prospective, multicenter randomized trial.

In conclusion, Cbl-b expression clearly indicated unfavorable prognosis and might be adopted as a novel prognostic marker in patients with resectable PDAC. Combining Cbl-b expression with serum CA19-9, pN category, and histological differentiation gave more precise prognostic information in PDAC patients, and might help to identify patients in need of much more stringent postoperative follow up. Detecting Cbl-b expression might assist clinicians in the choice of optimal treatment and postoperative management of PDAC patients.

## MATERIALS AND METHODS

### Patients and tissue samples

A total of 182 consecutive patients with resectable primary pancreatic carcinoma were treated at the Shengjing Hospital of China Medical University between January 2009 and February 2012. The diagnosis was confirmed by histopathology. Patients with intraductal papillary mucinous adenocarcinoma, mucinous carcinoma, malignant endocrine tumors, adenosquamous carcinoma, squamous carcinoma, and acinar cell carcinoma were excluded. Patients with a second cancer with a life-threatening phenotype (e.g., gastric stromal tumor, renal clear cell carcinoma, adrenocortical adenocarcinoma, gallbladder adenocarcinoma, and ovarian mucinous cystadenocarcinoma), patients died in the hospital within 30 days after surgery, and those with incomplete clinicopathological data were also excluded. The remaining 134 patients with PDAC were included in the study. None had received chemotherapy or radiation therapy prior to surgery. All patient data were retrospectively reviewed after the Research Ethics Committee of Shengjing Hospital of China Medical University approved the study.

Patient age, sex, location of the tumor, type of resection, tumor size, histologic differentiation, margin status (pancreatic resection, biliary, posterior, retroperitoneal, and mesenteric margins), tumor stage, node stage, vessel invasion, vascular tumor thrombus, invasion of adjacent organs (bile duct, duodenum, stomach, colon, jejunum, and spleen), tumor-node-metastasis (TNM) stage according to the seventh American Joint Committee on Cancer (AJCC) TNM system criteria, and preoperative serum CA19-9 level. Patients or their relatives and clinicians were followed up until 31 March 2016 by telephone interview to monitor survival or document the day of death. Death was confirmed from official local government death certificates or medical agencies, or by information obtained from family members. The medical records were independently reviewed by a physician to confirm the cause of death. A standard regimen of intravenous gemcitabine at a conventional dose and schedule was administered as postoperative adjuvant chemotherapy to patients who were willing and able to tolerate it, regardless of margin status or tumor stage.

### Immunohistochemistry (IHC)

PDAC tissue sections were deparaffinized, rehydrated, and washed in phosphate buffered saline (PBS) using an S-P immunohistochemistry kit (Fuzhou Maixin Biological Technology Ltd., Fujian, China) following the manufacturer’s instructions. Then slides were incubated with mouse anti-human Cbl-b (Santa Cruz Biotechnology, CA, USA) primary antibody in PBS at 4°C overnight in a humid box. After washing in PBS, the slides were incubated with secondary antibody, and the immune complexes were stained with 3,30-diamino-benzidine tetrahydrochloride (DAB; Fuzhou Maixin Biological Technology Ltd., Fujian, China) following the kit manufacturer’s instructions. Finally, the slides were counterstained with hematoxylin. Pre-immune rabbit serum at the same dilution was used as a negative control. Slide was evaluated by scanning the entire tissue specimen at low magnification (×10) and staining was confirmed at high magnification (×20 and ×40). Tumors with < 10% Cbl-b stained cells were considered low expression; those with ≥ 10% Cbl-b–stained cells were considered high expression. Two pathologists independently scored the slides.

### Statistical analysis

Data analysis was performed using SPSS software version 13.0 (SPSS Inc., Chicago, IL, USA) and R 3.3.1 software (http://www.R-project.org). Categorical variables were reported as numbers and percentages. Median values were used as cutoffs to categorize patient clinicopathological variables, and comparisons of Cbl-b expression were performed with the two-tailed *x*^2^ or Fisher’s exact test. The upper limit of the normal reference value (37 U/mL) was used as the cutoff for CA19-9; and for the nomogram, the group of CA19-9 ≥37 U/mL was also divided into another 3 groups: the CA19-9 values were sorted from low to high, and the values of 33% and 67% were used as the cutoff points. Overall survival (OS) was defined as the interval between the date of the surgery and either the date of death from any cause or the last follow-up visit. Survival curves were calculated by the Kaplan–Meier method, and the significance of differences in survival were determined by the log-rank test. A multivariate Cox proportional hazards model (forward selection) was fitted using all clinical and pathological variables, and risk factors identified in the multivariate analysis were used to construct a nomogram. The C-index and the AIC were used to evaluate the discrimination of the nomogram to predict prognosis. The higher the C-index, or the lower the AIC, the more accuracy was the prognostic prediction. Calibration curves were drawn to compare the relationship between observed and predicted responses. Two-sided *P*-values <0.05 were considered statistically significant.

## SUPPLEMENTARY MATERIALS FIGURE



## Abbreviations

Cbl-b: Casitas B-lineage lymphoma b
PDAC: pancreatic ductal adenocarcinoma
pT: pathological primary tumor
pTNM: pathological TNM
pN: pathological regional lymph node
OS: overall survival
CA19-9: carbohydrate antigen 19-9
CI: confidence interval
C-index: Harrell’s concordance index
AIC: Akaike information criterion
TKB: tyrosine kinase binding
NK: natural killer
EGF: epidermal growth factor
TNM: tumor-node-metastasis
AJCC: American Joint Committee on Cancer
IHC: immunohistochemistry
PBS: phosphate buffered saline
C-index: concordance index.
