# Supplementary material for: Cbl-b predicts postoperative survival in patients with resectable pancreatic ductal adenocarcinoma
Source: Oncotarget. 2017 Jun 27;8(34):57163–73. doi: 10.18632/oncotarget.18714 (PMC5593633; doi:10.18632/oncotarget.18714)
Supplement: Supplementary file 1 [file oncotarget-08-57163-s001.pdf]

Cbl-b predicts postoperative survival in patients with resectable pancreatic ductal adenocarcinoma

SUPPLEMENTARY MATERIALS

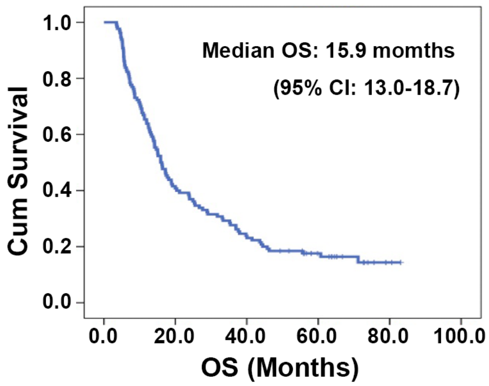

Supplementary Figure 1: Overall survival (OS) of all enrolled patients. Median OS was 15.9 months (95% CI = 13.0–18.7).
